# Using Google Scholar to track the scholarly output of research groups

**DOI:** 10.1007/s40037-019-0515-4

**Published:** 2019-05-17

**Authors:** Brent Thoma, Teresa M Chan

**Affiliations:** 1 Department of Emergency Medicine, University of Saskatchewan, Saskatoon, Saskatchewan Canada; 2 Division of Emergency Medicine, Department of Medicine, McMaster University, Hamilton, Ontario Canada; 3 McMaster program for Educational Research, Innovation, and Theory, McMaster University, Hamilton, Ontario Canada

**Keywords:** Research group, Google Scholar, Research impact

## Abstract

**Introduction:**

It is often necessary to demonstrate the impact of a research program over time both within and beyond institutions. However, it is difficult to accurately track the publications of research groups over time without significant effort. A simple, scalable, and economical way to track publications from research groups and their metrics would address this challenge.

**Methods:**

Google Scholar automatically tracks the scholarly output and citation counts of individual researchers. We created Google Scholar profiles to track the scholarly productivity of five research groups: an institutional educational research program, a division of emergency medicine, a department of emergency medicine, a national educational scholarship working group, and an international organization dedicated to online education. We added the publications of each group member to their respective group Google Scholar profile and a junior faculty member monitored the citations that were suggested.

**Results:**

Google Scholar tracked a diverse collection of five research groups over 6–36 months. In addition to having different organizational structures and purposes, the groups varied in size, consisting of 8–60 researchers, and prolificacy, with group citation counts between 1006–58,380 and group h‑indexes ranging from 19–101.

**Discussion:**

We anticipate that as this innovation becomes better known it will increasingly be adopted by traditional and non-traditional research groups to easily track their productivity and impact. Additional initiatives will be needed to standardize reporting guidelines within and between institutions.

**Electronic supplementary material:**

The online version of this article (10.1007/s40037-019-0515-4) contains supplementary material, which is available to authorized users.

## Introduction

Tracking the publications of a group of scientists (e.g. in an academic department, lab group, research collaborative, or institution) over time can be difficult [1]. Productive groups have numerous authors who publish frequently. Faculty, post-doctoral students, and graduate students move between institutions, muddying amalgamated metrics. Authors may not submit their latest publications on a regular basis resulting in missing metrics.

However, tracking these metrics is important to research groups who need to justify their use of resources, demonstrate the growth of their research programs over time, and promote the success of their research program [2]. As a result, several methods have been developed to track publication metrics. For example, some academic departments have their researchers submit their curricula vitae (CVs) annually. While this results in a comprehensive database, it is resource intensive to pull publications from CVs into a central registry. Digital solutions include institutional subscriptions to citation indices such as Scopus (Elsevier) and Web of Science (Clarivate Analytics) [3]. However, they cost money to access and do not make their results publicly available. Further, while they can track and amalgamate the publication metrics of individual faculty, they have more difficulty tracking research groups that are more ambiguous. ResearchGate provides a free service that can be used by research or project groups; however, its citation tracking is incomplete (it is limited to papers that have been uploaded to ResearchGate) [4].

Google Scholar has been used successfully by individual researchers to track their scholarly output and citations and is thought to be as good as many other search engines as a source of bibliometric data [5]. Notably, once papers have been added to a profile, it is able to identify new publications by the same researchers. Although Google Scholar has been criticized for being over-inclusive, it is becoming an accepted academic standard [5]. Here, we aim to determine whether it is feasible for a variety of research groups to use Google Scholar profiles to track their scholarly output.

## Methods

We created Google Scholar profiles for five types of research groups: the members of an educational research program (the McMaster program for Education Research, Innovation and Theory), the faculty of an academic division (the Division of Emergency Medicine at McMaster University), the faculty of an academic department (the Department of Emergency Medicine at the University of Saskatchewan), the members of a national working group (the Education Working Group (EWG) of the Canadian Association of Emergency Physicians (CAEP)), and the editors of an international online education website (Academic Life in Emergency Medicine (ALiEM), https://aliem.com/). These five groups were intentionally selected to range broadly in terms of size (from small to large) and ambiguity (from a defined department to a relatively poorly defined working group). Institutional Review Board/Research Ethics Board approval was not sought as this work met the criteria for exemption under performance review. Further, only publicly available data were utilized.

**Fig. 1:**
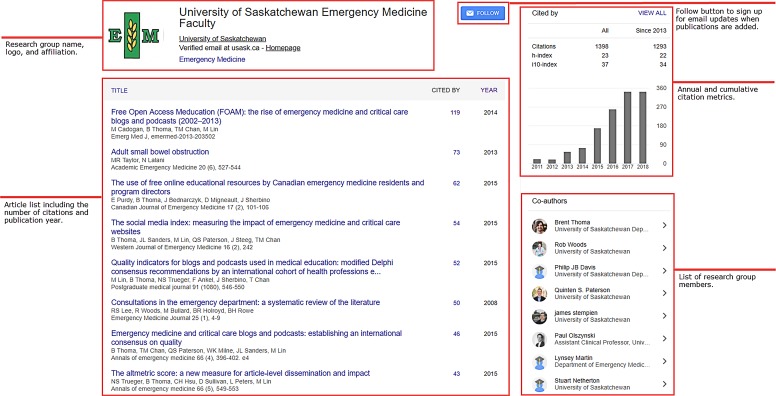
Labelled Google Scholar profile of the University of Saskatchewan Department of Emergency Medicine as of 24 July 2018

A labelled outline of a group Google Scholar profile is provided in Fig. 1 while a screencast demonstrating the creation of a group profile is available as a Video on the journal website. In short, Google email (Gmail) addresses were created for each account using an institutional email address. The authors developed a list of members for each research group and searched for their research on Google Scholar. Each member’s research was found on Google Scholar and added to the group profile. To prevent the addition of inappropriate papers and metrics to the accounts, the Google Scholar settings were set to require approval from an account administrator before adding new papers to the publication list. Criteria for the appropriateness of an article were determined by the function of the group (e.g. all medical articles were included for the division/department, but only medical education articles were included for the education scholars group).

The outcome of interest was the feasibility of setting up group Google Scholar profiles for various research groups. The quantitative characteristics of each research group were collected to demonstrate their diversity. Qualitative descriptions were used to describe the feasibility and challenges encountered in the set-up and maintenance of the research groups.

## Results

We were able to set up and begin populating new Google Scholar accounts for each research group (Table 1) within minutes. When a member of the research group had a pre-existing, up-to-date Google Scholar profile, adding their publications to the group profile page was as simple as identifying it. When a member of the research group did not have a pre-existing Google Scholar profile, it was equally quick to find a publication list, but it had to be vetted with more care. After adding roughly 10 papers by a given author, the Google Scholar algorithms were able to accurately suggest additional updates over time. The groups varied in size (8–60 researchers) and publication history (citations ranged from 1006 to 58,380; h‑index ranged from 19–101).

**Table 1:** Group Google Scholar Profile data as of 5 August 2018

Type of group	Tracking since	Institution or group	Number of scholars	Total citations	Group h‑index
Academic division	September 2015	McMaster University Division of Emergency Medicine	33	12,590	50
Academic department	August 2015	University of Saskatchewan Department of Emergency Medicine	11	1411	23
Research centre	November 2017	McMaster Program for Education Research, Innovation, and Theory	8	58,380	101
Online education organization	October 2017	Academic Life in Emergency Medicine	15	1006	19
National working group	January 2018	Canadian Association of Emergency Physicians Education Scholars Group	60	13,035	53

Due to its size and scope, the creation of the profile for the CAEP Education Scholars Group [6] provided the greatest challenge. It included 60 researchers from multiple institutions and many of them had never published with each other before. However, even in this case it was possible to create and populate the Google Profile page in less than an hour. Given the large volume of articles and the focus of the group on medical education scholarship, it took another 2 hours to review the articles suggested for the members to determine whether they were appropriate for inclusion.

After the groups were created, the scholarly output of the research groups was tracked for 6–30 months. Each account was managed by a single junior faculty member who reviewed the auto-updates for a few minutes every 1–2 months. Human oversight was required to ensure that the suggested additions were accurate, particularly if a group member had a common name. It was helpful for the junior faculty member reviewing the suggested additions to have knowledge of the individuals within the group and their research programs as this allowed them to include and exclude articles quickly.

## Discussion

While the creation of group Google Scholar profiles has been described in guides online as early as 2013 [7], this is the first report that we are aware of in the published literature that demonstrates the potential and versatility of this innovation. Given the ease of set-up and tracking we anticipate that, if the ability to create group profiles were more widely known, traditional and non-traditional academic groups would increasingly create profiles to quantify their team’s scholarly productivity. These profiles may be particularly useful to small or poorly resourced research groups or institutions, medical education centres which draw faculty from numerous departments, and research entities with faculty disseminated across institutions.

There are multiple potential benefits to this beyond tracking the growth of group publications and citation metrics [2]. First, the creation of a group Google Scholar page can serve to advertise the success of a group in a publicly accessible way. Second, it provides a relatively comparable way to contrast the research productivity of various groups. Third, other scholars interested in the work of a group will be able to find it without searching between authors and journals over years. Fourth, new research published by a group could be easily tracked by signing up for publication alerts from its Google Scholar profile. Finally, we hypothesize that tracking the collective research output of a group (as opposed to an individual) is likely to foster collaboration and the pursuit of collective goals. For example, the Department of Emergency Medicine at the University of Saskatchewan uses its Google Scholar profile to track the productivity goals in its strategic plan, direct trainees to faculty conducting research in various areas, and compare its progress to related groups such as the Division of Emergency Medicine at McMaster University.

As outlined in Table 1, not all groups are the same and interpretations of the collective scholarly output should be made only while considering the context (e.g. number of researchers, field of research, mission of the group) of a given group. At the very minimum, the number of scholars within a group should be taken into consideration. For instance, it may be worth pursuing some sort of comparative metric that allows for some element of adjustment for the number of scholars within a given group. To compare groups, new metrics will be required to adjust for various elements that can skew the data. For instance, a single eminent scholar with many citations may mask the lower performance of new faculty or an entire group. Finally, Google Scholar is but one method by which one might quantify bibliometric contributions and productivity. There are many types of scholarship that simply will not be well captured via bibliometric measures—those leaders looking to encourage curriculum design for teachers or quality improvement in their hospitals may find these metrics limited in their ability to capture these newer forms of academic health sciences scholarship. A judicious examination of one’s own group that aligns outcome measures with desired productivity would be optimal for fostering the success of all [8].

## Conclusions

While research metrics have been tracked for decades, Google Scholar profiles can provide a wide variety of research groups with an easy, simple, free, and scalable solution to the problem of tracking their collective scholarly output. With broader adoption of these techniques it will become increasingly possible to quantify and compare the productivity of a variety of types of scholarly organizations.

## Caption Electronic Supplementary Material

Video 1. Video guide to creating a group Google Scholar profile
